# Online validation of combined mood induction procedures

**DOI:** 10.1371/journal.pone.0217848

**Published:** 2019-06-04

**Authors:** David Marcusson-Clavertz, Oscar N. E. Kjell, Stefan D. Persson, Etzel Cardeña

**Affiliations:** 1 Department of Psychology, Lund University, Lund, Sweden; 2 Department of Psychology, Ludwig Maximilian University of Munich, Munich, Germany; Temple University, UNITED STATES

## Abstract

Film clips, music, and self-referential statements (termed *Velten*, after their originator) have been successfully used to temporarily induce sadness and happiness. However, there is little research on the effectiveness of these procedures combined, particularly in internet-based settings, and whether Velten statements contribute to alter mood beyond the effect of simple instructions to close one's eyes and enter the targeted mood. In Study 1 (*N* = 106) we examined the effectiveness 80 Velten statements (positive, negative, neutral-self, neutral-facts) to create brief and effective sets that might be used in future research. In Study 2 (*N* = 445) we examined the effect size of 8-min combined mood induction procedures, which presented video clips in the first half and music excerpts with Velten statements or closed eyes instructions in the second half. Participants answered questionnaires on social desirability, joviality, and sadness before being randomly assigned to 1 of 7 groups varying in Valence (positive, negative, neutral) and Velten (closed eyes control, self-referential Velten, and, in the case of neutral condition, factual statements). Subsequently, participants completed the joviality and sadness scales a second time. Compared to the neutral conditions, the positive mood inductions increased joviality (Hedges *G* = 1.35, 95% CI [1.07, 1.63]), whereas the negative mood inductions increased sadness (Hedges *G* = 1.28, 95% CI [1.01, 1.55]). We did not observe any significant difference between Velten and closed eyes instructions in inducing joviality or sadness, nor did we observe any significant difference between neutral Velten statements referring to self and facts. Although social desirability bias was associated with reports of greater joviality and lower sadness, it could not account for the effects of the positive and negative mood induction procedures. We conclude that these combined mood induction procedures can be used in online research to study happy and sad mood.

## Introduction

People’s moods vary at different times. For instance, about 50% of variance in negative affect in everyday life is due to within-person factors, including variation across moments and days [1]. As daily life variability in affect predicts important health-related behaviors (e.g., sleep [2]), it is important to understand temporary fluctuations in affect. A common experimental approach to study mood is to employ mood induction procedures (MIPs) in which experimental stimuli are administered in a controlled fashion to alter people’s mood temporarily. Widely used examples of MIPs include presenting affectively-laden video clips, songs, or self-referential statements known as Velten statements [3]. A meta-analysis indicated that MIPs on average produce about a one standard deviation change in mood compared to control procedures studies [4]. However, there is substantial heterogeneity of effect sizes across studies, and a recent meta-analysis on internet-based studies failed to find support for the effectiveness of happiness MIPs administered online [5], which may be due to the diversity of MIPs. Several reviews have recommended video clips as they induce medium-to-large changes in positive and negative mood [3–5], but a handful of studies have observed large effect sizes for *combined* MIPs (e.g., reading Velten statements while listening to music; [4]). In this paper, we developed and tested combined MIPs administered online comprising video clips followed by music excerpts with Velten statements or closed eyes instructions.

### Velten induction

In 1968, Emmett Velten Jr. examined whether semantic content could influence people’s mood. He developed self-referential sentences and asked participants to try to feel the mood suggested by the sentences, either positive (e.g., *This is great—-I really do feel good—I am elated about things*) or negative (e.g., *I have too many bad things in my life*). Participants were then tested on seven outcomes, including self-reported mood, writing speed, decision time, and number of spontaneous verbalizations during the test [6]. As a control condition, he asked participants to read statements intended to be neutral (e.g., *Utah is the Beehive State*). The results were generally in the expected direction with, for instance, those receiving the positive mood induction reporting more positive mood, writing more words per minutes, and producing more spontaneous verbalizations during the test than the other groups (but there were no significant differences for a perceptual ambiguity outcome). Since then, self-referential and suggestive of a targeted mood statements (typically called Velten statements), have been among the most commonly used and successful techniques to influence mood [7, 8] and been extended to various states like dissociation [9]. One reason for its popularity may be its ease to standardize and administer, which has allowed it to be successfully combined with music [10]. The duration of the induction can also be easily manipulated by changing the number of statements and their duration. Successful induction has been shown with as few as 12 statements [11].

Critics have argued that the technique is susceptible to demand characteristics [7] and social desirability biases [12]. Tests of demand characteristics have yielded mixed results [6, 13], and others have argued that demonstrating that individuals can simulate a changed mood when instructed to do so does not necessarily show that they will pursue such simulations when not asked to [14]. Although previous research has failed to demonstrate a role of social desirability biases in responding to Velten MIPs [15, 16], these studies have not evaluated a positive mood induction. Another issue with Velten MIPs is that positive and negative Velten statements are self-referential, whereas neutral Velten statements are not, thereby conflating valence with self-reference. However, activating self-referential thoughts about personal relationships and similar themes might increase the tendency to mind wander [17], and as such, Velten statements might influence cognitive outcomes due to self-reference rather than affect. For situations like this, we set out to design neutral statements that are self-referential and more comparable with positive and negative statements.

Despite having been the most frequently used MIP [4], previous research has questioned whether Velten MIPs are more effective than just simple instructions to enter the target mood. One study tested four conditions (Velten, Music, Velten + Music, or Instructions only; 15 individuals per condition) to enter anxious and sad moods [18]. All conditions successfully altered moods, but the study did not find any significant difference between the conditions. One possibly mitigating factor for Velten MIPs is that the statements themselves might vary in effectiveness, but they have been rarely independently tested rather than evaluated as a complete battery. One exception, particularly relevant for study 1, was a study asking undergraduates to rate each of 84 statements [19]. Jennings and colleagues [19] tested 25 statements per condition (positive, neutral, negative), of which most statements had been used previously [20]. Participants were asked to try to experience the mood suggested by each of the 84 statements and then rate the mood on a 9-point valence scale. After rank-ordering the valence ratings of these statements, the authors examined whether positive statements were placed in the top-third, the negative statements in the bottom-third, and the neutral statements in the middle third. They found that merely 52 statements were ranked in the expected third (i.e., 62% of the statements were given ratings congruent with their designated category). This suggests that further refinement of Velten statements is needed to optimize the procedure before comparing it with other techniques, such as simple instructions to get into the targeted mood.

## Study 1

Our aim was to create short Velten forms that could be used to quickly induce temporary changes in happiness and sadness and to develop and evaluate a set of neutral self-referent statements that could be used as a control condition. Our goal was to extract at least 15 statements with high positive valence ratings, 15 negative statements with high negative valence, and 15 neutral-self and 15 neutral-facts close to each other and to the midpoint of the bipolar valence scale.

### Method

#### Participants

Out of the 121 participants that completed the study, 15 were excluded for answering the control questions incorrectly (see Materials). Of the remaining 106, there were 65 females, 40 males, and 1 person with a missing response. The average age was 35.44 years old (*SD* = 11.17). Participants completed the survey using laptops (*n* = 53), desktop computers (*n* = 36), smartphones (*n* = 11), or tablet computers (*n* = 6).

### Materials

We included 80 Velten statements. divided into four equally large categories (positive, negative, neutral-facts, neutral-self). Participants were asked to: 1) read each statement carefully and, if applicable, try to experience it as though it were happening to them; and 2) rate how they felt while reading the statement. The scale ranged from 1 (*very unhappy*) to 9 (*very happy*) and was coupled with nine Self-Assessment Manikin (SAM) figures. We derived the items from two sources [19, 21] (hereafter referred to as the Jennings et al. and Lessard studies), except for the neutral-self statements, which we developed ourselves (see Table 1).

**Table 1:** Valence ratings of 20 neutral-self statements. Asterisk (*) Denotes Statements Included in the Mood Induction Procedures in Study 2.

	Name	*M*	*SD*
1*	From time to time I use the internet for finding information	6.52	1.36
2*	I occasionally watch documentaries	6.30	1.41
3*	I buy groceries just like everyone else	6.10	1.45
4*	If I think about it, things tend to even out for me	6.03	1.17
5*	I go to the convenience store sometimes	5.86	1.33
6*	Everyone seems to be going about their everyday routine just like me	5.73	1.26
7*	Sometimes my life is just pretty normal	5.59	1.30
8^a^	Today is one of those days where things go pretty much as expected, neither better nor worse	5.51	1.29
9*^b^	I read the newspapers every once in a while	5.50	1.52
10*	This is a normal day, just like any other	5.47	1.21
11*	Today is just an ordinary day	5.37	1.37
12*	Some days are neither good nor bad	5.25	1.16
13*	I feel pretty neutral today	5.20	1.18
14*	Some of my interactions with people are just pretty average	5.15	1.20
15*	Lately, day-to-day life has been pretty ordinary for me	5.13	1.32
16	Almost every day I do commonplace things like chores	5.08	1.55
17	Everyday tasks like brushing my teeth are unremarkable	5.01	1.34
18	I feel neither great nor terrible, just average	4.97	1.07
19*	My energy can fluctuate from one day to the next	4.79	1.52
20	I don't feel very peppy or sluggish, just OK	4.74	1.06

The ratings of the other 60 statements (positive, negative, neutral-facts) can be obtained from the corresponding author. Sample sizes vary between 104–106 individuals due to missing responses.

^a.^ We did not include this statement in Study 2 to reduce the number of characters and make the conditions more similar in terms of character length.

^b.^ This item was revised to “I check the news every once in a while” in Study 2 to make it more inclusive.

We included 20 positive statements, 18 of which were derived from the Jennings et al. study [19] and 2 from Lessard [21] (e.g., *If I set my mind to it*, *I can make things turn out fine*). We excluded four items from the Jennings et al. study based on negative feedback we received during piloting (*My future is so bright I’ve got to wear shades; I feel completely aware; Nothing can bum me out now; When it comes right down to it*, *I’m just too cool*).

We included 20 negative statements of which 17 were derived from the Jennings et al. study [19] and 3 were revised statements from Lessard [21] (e.g., *I've doubted that I'm a worthwhile person*). We excluded four items used in the Jennings et al. study because they referred negatively to “trying” (which might induce demand characteristics not to make an effort on subsequent tasks; *I'm tired of trying*; *What's the point of trying*?) or we deemed them too despairing (*I'm completely alone; There is no hope*).

We included 20 Neutral facts from the Jennings et al. [19] study. Three of these were edited from referring to episodes (*Elephants carried the supplies; The rug was made according to an old Navajo pattern; Mules hauled the supplies up the mountain*) to factual statements (*Elephants are large mammals; Typical Navajo textiles have geometric patterns; Mules can carry goods on their back*).

We developed a set of 20 items intended to be self-referential and induce a neutral mood characterized by low sadness and low happiness so that they could be compared to positive and negative MIPs without confounding valence and self-reference. Our purpose was to identify at least 15 satisfactory items that could be used in future MIP research. As a pilot, we assessed a preliminary set of 25 neutral-self items (together with 25 positive and 25 negative items from the Jennings et al. and Lessard studies [19, 21]) by asking a panel of four psychologists to evaluate item clarity and effectiveness of inducing the intended mood and suggest improvements. We then revised the items and selected 20 items per set and tested them in Study 1 (for the novel neutral-self statements, see Table 1).

We included two intermittent control items among the Velten statements. Participants were asked to “Select alternative 3 here” for control item 1 and “Select alternative 7 here” for control item 2. This approach has been shown to increase reliability [22].

#### Procedure

Volunteers at Academic Prolific were invited to rate the emotional valence of 80 statements. The statements were presented in random order in 10 pages; with two intermittent control items (item 1 at the first page and item 2 at the seventh page). At the end, participants indicated their gender, age, and what device they were using to complete the study, before they were compensated with £1.25 each. This research was approved by our regional ethics board (Regionala Etikprövningsnämnden, Lund, Sweden).

### Results and discussion

Participants’ ratings of each neutral-self statement are summarized in Table 1 (Ratings of statements from the other categories can be obtained by request to the corresponding author). Like Jennings et al. [19], we first examined the congruency of our *a priori* categorization with participants’ valence ratings, by counting the number of statements from the positive category that ranked among the top 25%, negative statements in the bottom 25%, and neutral statements in between, respectively. There were 18 (out of 20) statements from the positive category that were ranked in the top quarter in valence, whereas all 20 statements from the negative category ranked in the bottom quarter as expected. From top-to-bottom-quarters, the two neutral categories were ranked in the following way: 1 neutral-self and 1 neutral-facts statements were ranked in the top quarter; 7 neutral-self and 11 neutral-facts in the second quarter; 12 neutral-self and 8 neutral-facts in the third quarter; and 0 of each in the bottom quarter. Thus, except for two positive, one neutral-self, and one neutral-fact, the statements were ranked in the expected quarters.

As for the average ratings, the mean valence was 6.84 (*SD* = 1.30) for the positive statements, 5.74 (*SD* = 1.27) for the neutral-facts, 5.41 (*SD* = 1.26) for the neutral-self, and 3.15 (*SD* = 1.53) for the negative statements. Compared to the valence ratings of the neutral-facts statements, there was a large increase for the ratings of positive statements, *t*(103) = 6.22, *p* < .001, Hedges G_av_ = 0.85, a very large decrease for the negative, *t*(102) = -16.44, *p* < .001, Hedges G_av_ = -1.83, and a small decrease for the neutral-self statements, *t*(103) = -2.18, *p* = .032, Hedges G_av_ = -0.26. That is, although neutral-self statements were rated above the midpoint (5) of the scale, these scores were weakly, but significantly, lower than those for the neutral facts statements. To conclude, the ratings were largely congruent with the designated categories and the differences between the valence categories were large. We therefore decided to use the more effective items for the MIPs in Study 2.

## Study 2

As internet-based MIPs have been inconsistent in inducing the targeted moods [5], whereas laboratory-based MIPs that combine various induction techniques have yielded large effect sizes [4], we aimed to test combined MIPs online. In addition to seeking MIPs that could yield relatively large effects online, Study 2 tested whether Velten or closed eyes instructions were more effective when being presented with music following a video clip. We selected 60 Velten statements based on the ratings in Study 1. We reasoned that by selecting effective Velten statements based on Study 1 ratings, it would be informative to follow up on previous research [18], which did not find any significant difference between Velten and simple instructions to get into the targeted mood. We expected that positive MIPs would increase happiness and negative ones would increase sadness, but did not expect any changes in happiness or sadness from the Neutral MIPs or any differences between Velten and closed eyes. We pre-registered the study at aspredicted.org (https://aspredicted.org/dc7id.pdf).

### Method

#### Participants

Out of the 699 individuals that opened the online survey, 500 completed the study. Of those 500 individuals, 51 failed to correctly answer all control items (this was mainly due to 35 participants having missing responses on the first control item) and were excluded as pre-registered. An additional four participants were identified as multivariate outliers (Mahalanobis distances, *p* < .001) and excluded so that the final *N* includes 445 individuals. Of them, there were 224 females, 220 males, and 1 person with a missing response. The average age was 35.94 years old (*SD* = 12.25). Participants completed the survey using desktop computer (*n* = 152), laptop (*n* = 287), tablet computer (*n* = 5), or smart phone (*n* = 1).

To measure attrition as a function of induction, we measured how many individuals started the induction for each group and compared these numbers with the final sample sizes after excluding those who failed any control item or were identified as outliers. The final sample included 86% out of those who started the Positive Closed eyes induction, 89% for Positive Velten, 89% for Negative Closed eyes, 84% for Negative Velten, 84% for Neutral Closed eyes, 86% for Neutral Velten-Self, and 81% for Neutral Velten-Facts. We did not observe a significant difference in attrition rate across the seven groups, χ(6) = 3.26, *p* = .776.

#### Materials

We used two subscales from the self-report measure Positive and Negative Affect Schedule–Expanded (PANAS-X; [23]). The PANAS-X is an expanded form of the 20-item PANAS [24]. As positive and negative affect comprise distinct, but related, subcomponents such as fear, hostility, and guilt [24, 25], we selected the two scales we expected the MIPs would alter the most (joviality, sadness). The Joviality scale includes eight items (happy, joyful, delighted, cheerful, excited, enthusiastic, lively, energetic), whereas the Sadness scale includes five items (sad, blue, downhearted, alone, lonely). Across 11 samples, the median reliability estimate (Cronbach’s α) was .93 for Joviality and .87 for Sadness ([23].

The instructions prompted participants to “Please answer honestly how you feel right now.” For each item, participants were asked to rate how they felt using a Likert scale with the following labels: 0 (*very slightly or not at all*), 1 (*a little*), 2 (*moderately*), 3 (*quite a bit*), 4 (*extremely*). The mean score of Joviality was used to index positive mood and the mean score of Sadness was used to index negative mood.

The Patient Health Questionnaire-2 (PHQ-2) screening form measures depressed mood over the past two weeks [26]. It includes two items (*Little interest or pleasure in doing things*; *Feeling down*, *depressed or hopeless*) answered on a scale including 0 (*Not at all*), 1 (*Several days*), 2 (*More than half the days*) and 3 (*Nearly every day*). Those who answered 0 or 1 on both items passed screening criteria.

The revised ***Social Desirability Scale (SDS)***, which excludes an item on drug use, consists of 16 items and has shown good psychometric properties in previous studies [27], including a Cronbach’s alpha of .80 and a convergence correlation of *r* = .69 with the Marlowe-Crowne scale, another scale measuring social desirability [28]. In further support of its validity, the SDS is sensitive to instructions presumed to increase demand characteristics (i.e., imagining applying for a job; [27]). Participants are instructed to read each statement carefully and decide if that statement describes them or not by answering “True” or “False.” There are 6 items in which the socially desirable response is designated to be “true” (e.g., “I always eat a healthy diet”) and 10 items in which the socially desirable response is “false” (e.g., “I occasionally speak badly of others behind their back.”). Socially desirable responses are coded as 1, and we used the mean score on the SDS in the analyses to include participants with missing responses.

All MIP conditions were 8 min long and consisted of a 4 min video clip that included dialogues and music, followed by a 4 min extract of instrumental music. The music was accompanied by instructions to either keep the eyes closed (closed eyes condition) or reading Velten statements, in which the statements were either self-referential (e.g., “I feel worthless”) or referred to facts (e.g., “New York City is in New York state”).

The *positive MIP* begins with instructions to get into a *happy* mood, followed by a 4 min video clip showing a musical piece called Hakuna Matata from the animated motion picture the Lion King [29]. The clip begins with characters Timon and Pumba explaining to Simba the concept of “Hakuna Matata,” which means “No worries.” Then they sing the song for Simba who later joins in. In addition to the singing, the clip includes some comedic scenes in which Timon and Pumba teach Simba how to eat like they do. The clip ends when the song fades out as the three of them are walking towards the horizon. A Hakuna Matata clip has previously been used successfully to induce happiness [30].

After the video clip, participants were asked to listen to the first 4 min of the instrumental piece “Coppélia, Act I: 1. Prélude et Mazurka,” composed by Léo Delibes. This music is frequently used in MIP studies to induce positive affect[31]. For instance, it has been successfully used to increase happy mood compared to neutral music [32]. The music was accompanied by either closed eyes instructions or Velten statement. We selected the 15 Velten statements with the highest positive valence from Study 1.

The *neutral MIP* begins with instructions to get into a *neutral* mood, followed by a 4 min clip about Magnets edited from the documentary program Modern Marvels, season 8 episode 35 [33]. We chose this clip because documentary materials have been previously used to induce neutral moods, although they may increase boredom [34]. Given the popularity of Modern Marvels and its fast editing, we assumed that it would induce lower levels of boredom than previously used documentary clips. The 4 min clip comprises four parts of the episode. It begins with the vignette in which the concept of Magnets and some of its applications are introduced (40s). It cuts to a visual demonstration of a hand holding a magnet above various objects, showing that it attracts some items and repels others, while magnets are defined (14s). It then cuts to a visual demonstration of a magnetic field in which a scientist puts a piece of paper above a magnet, and then pours iron powder over the paper to show how they circulate around the magnet (50s). It then cuts to an animation in which electrons circulate around an atom to form a magnetic field (50s). It finally cuts to a description of the discovery of the lodestone, a naturally magnetized piece of magnetite, briefly discussed by author James D. Livingstone followed by a demonstration of an early application of magnets, the compass (1 min 27s).

After the video clip, participants were asked to listen to the first 4 min of the instrumental piece “Variations for Winds, Strings and Keyboards” by Steve Reich. In a study asking 27 participants to rate various compositions on a valence scale, 24 out of 27 rated this piece as neutral by giving it a middle point score of 5 on a 10-point mood scale [35]. The music was accompanied by either closed eyes instructions or Velten statements. To minimize the differences between self-referential and factual Velten statements, we selected the 15 Velten-self statements with the highest positive valence (see Table 1) and the 15 Velten-facts statements with the lowest valence.

The *negative MIP* begins with instructions to get into a *sad* mood, followed by a 4 min clip showing the death of Mufasa from the animated cartoon the Lion King [29]. It begins with a red-billed hornbill, Zazu, flying above a wildebeest stampede to find a lion cub, Simba, in danger. Zazu alerts Simba’s father, Mufasa, who saves Simba. While saving Simba, Mufasa is drawn back into the stampede and struggles to get back up, calling his brother Scar for help. Scar instead pierces Mufasa’s palms, which leads to Mufasa falling back to the stampede. Soon after, Simba finds his father dead. Simba tries to wake his father up without success, calls for help but no one answers, and then starts to cry. He finally curls himself under his father’s arms. This film scene has been successfully used to induce sadness [36].

After the video clip, participants are asked to listen to the first 4 min of the piece “Adagio for Strings, Op. 11” by Samuel Barber. This music has been successfully implemented to induce sadness [37]. The music was accompanied by either closed eyes instructions or Velten statements. We selected the 15 Velten statements with the highest negative valence from Study 1.

#### Procedure

Participants who passed the PHQ-2 screening criteria could find the study on the Academic Prolific platform. After giving informed consent, participants were asked to complete the SDS, followed by the two PANAS-X subscales with the items presented in random order. Participants were randomized into seven MIP groups that included 4 min video clips followed by 4 min song clips with either Velten or closed eyes instructions. (i.e., Positive Velten, Positive Closed eyes, Negative Velten, Negative Closed eyes, Neutral Velten-Self, Neutral Velten-facts, Neutral Closed eyes). To mitigate the risk of demand characteristics, we provided the following instructions for all conditions:

Do not worry if you do not immediately feel the mood. It might take a while. We are trying different procedures and the same procedure will not suit everyone. However, this procedure has worked well on a lot of people. Please just try your best and answer honestly when we ask about your mood!

After the 8 min MIP, participants completed the two PANAS-X subscales with the items presented in random order again. Next, they answered some demographic questions, before completing a trait-like questionnaire on mind wandering tendencies (for exploratory purposes, not reported in this paper). Participants were then compensated, and those receiving the neutral or negative MIPs received a positive MIP to increase the likelihood that participants completed the study in a positive mood. This research was approved by a Swedish regional ethics board.

### Analyses

We computed effect sizes for pre vs. post differences using the Excel spreadsheet created by Lakens [38]. Specifically, we computed Hedges G_av_ and common language effect sizes. All other analyses were performed using SPSS version 20. For analyzing the effects of inductions across various groups, we performed repeated measures analyses of covariance (RM ANCOVAs) using the SDS as a covariate (as pre-registered). The Box’s *M* test of Homogeneity of the variance-covariance matrices was significant (*p* ≤ .001), but the sample sizes were relatively similar across the groups (the largest and smallest samples differed by a ratio of 1.65:1) so we report the raw results without corrections. If anything, the tests are conservative when there are larger variances in larger groups [39]. As there were potential univariate outliers in some analyses, we report the raw results with univariate outliers included but also note whether dropping them impacted the results notably. Our α was set to .05. The figures were created with R [40].

### Results

#### Summary of research measures

Skewness and kurtosis for the different scales were below 1.25. The PANAS measurements (pre, post) showed good reliability with Cronbach’s α of at least .87, whereas the Social desirability scale showed acceptable reliability (α = .70). Critically, the PANAS difference scores (measuring change in mood from pre to post-induction) showed high reliability across individuals (α = .92 for Joviality and α = .87 for Sadness). Social desirability was associated with Joviality pretest scores, *r*(443) = .29, *p* < .001, Joviality posttest scores, *r*(443) = .20, *p* < .001, and Sadness pretest scores, *r*(443) = -.14, *p* = .004. There was no significant association between Social desirability and Sadness posttest scores, *r*(443) = - .06, *p* = .172. These results indicate that individuals with greater tendency to report socially desirable behaviors also reported more positive mood states, at least prior to the induction.

#### Pre-induction

We first examined whether there was any difference across the seven groups prior to the induction. Table 2 shows the means across conditions pre and post-induction. The one-way ANOVAs did not yield any significant baseline difference between the seven groups in Joviality, *F*(6,438) = 0.42, *p* = .864, η_p_^2^ = .01, or Sadness, *F*(6,438) = 0.70, *p* = .652, η_p_^2^ = .01. Across all groups, participants rated their Joviality (*M* = 1.52, *SD* = 0.86) as significantly higher than their Sadness (*M* = 0.80, *SD* = 0.87, *t*[444] = 10.75, *p* < .001) prior to induction. As there were no significant differences prior to induction, we next analyzed the valence conditions (positive, negative, neutral) separately to address the three primary research questions and then analyzed them all together (except for the neutral Velten-facts group) to examine interactions between Velten-self and Valence conditions.

**Table 2:** Mood (sadness, joviality) by valence condition (negative vs. neutral vs. positive) and Velten group (closed eyes vs. Velten self vs. Velten facts).

Outcome	Condition	Group	Pre *M* (*SD*)	Post *M* (*SD*)	Paired *t*-test for Pre vs. Post	Effect Size (Hedges G_av_)
Sadness	Negative	Closed Eyes (*n = 74*)	0.76 (0.79)	1.80 (1.01)	*t*(73) = 11.13***	1.15
		Velten (*n = 68*)	0.76 (0.87)	1.51 (0.97)	*t*(67) = 5.98***	0.81
	Neutral	Closed Eyes (*n = 46*)	0.72 (0.84)	0.71 (0.95)	*t*(45) = -0.03	-0.00
		Velten Self (*n = 67*)	0.94 (0.91)	0.73 (0.74)	*t*(66) = -2.93**	-0.26
		Velten Facts (*n = 59*)	0.70 (0.71)	0.58 (0.60)	*t*(58) = -1.69	-0.17
	Positive	Closed Eyes (*n = 66*)	0.90 (1.03)	0.51 (0.82)	*t*(65) = -5.34***	-0.42
		Velten (*n = 65*)	0.82 (0.92)	0.42 (0.63)	*t*(64) = -6.10***	-0.52
Joviality	Negative	Closed Eyes (*n = 74*)	1.53 (0.84)	0.60 (0.67)	*t*(73) = -10.67***	-1.23
		Velten (*n = 68*)	1.57 (0.94)	0.64 (0.79)	*t*(67) = -11.44***	-1.07
	Neutral	Closed Eyes (*n = 46*)	1.64 (0.82)	1.33 (0.93)	*t*(45) = -2.70**	-0.35
		Velten Self (*n = 67*)	1.52 (0.88)	1.30 (0.86)	*t*(66) = -2.35*	-0.24
		Velten Facts (*n = 59*)	1.44 (0.79)	1.25 (0.81)	*t*(58) = -1.87	-0.23
	Positive	Closed Eyes (*n = 66*)	1.42 (0.83)	2.00 (0.87)	*t*(65) = 9.50***	0.68
		Velten (*n = 65*)	1.54 (0.90)	2.21 (1.01)	*t*(64) = 9.22***	0.70

All induction procedures included a 4-min video clip and a 4-min musical piece.

*** p ≤ .001

** p ≤ .01

* p ≤ .05

#### Do the positive mood inductions increase joviality?

**Fig 1:**
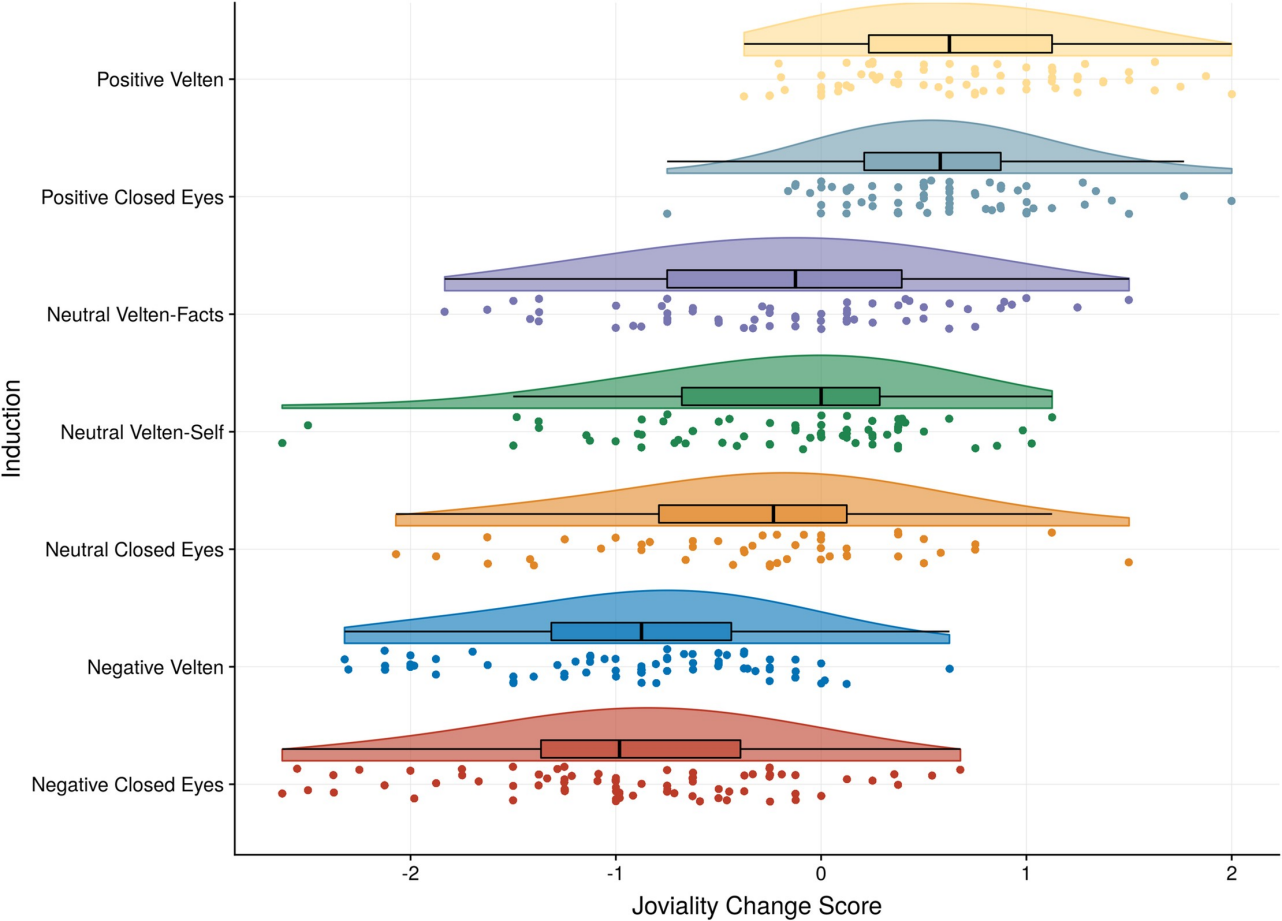
Illustration of joviality change scores across mood induction procedures. A raincloud plot showing the distributions of change scores in Joviality from pre to post-induction grouped by induction condition. The small dots below each raincloud show the observed scores of that condition (jittered). The lower and upper hinge on the boxplots represent the first and third quartile with the median in between, whereas the whisker lines extend to the most extreme values but no further than 1.5 × the interquartile range from the hinge. The raincloud shapes illustrate the distribution of observations based on Kernel density probability functions computed for each condition (scaled to have equal heights across conditions). To highlight “no change” from pre- to post induction, 0 is indicated with a vertical line.

The RM ANCOVA with the two positive Groups (Velten vs. Closed Eyes) as between subjects factor, Induction (pre vs. post) as within-subjects factor, and Social desirability as a covariate indicated a clear main effect of the Induction, *F*(1,128) = 17.90, *p* < .001, η_p_^2^ = .12, as expected (see Fig 1). The common language effect size indicates that the likelihood that a person rates higher joviality after the positive inductions than prior to it is 88%, and the effect size was between medium and large (Hedges G_av_ = 0.68, 95% CI [0.56, 0.82]). There was no significant interaction between Induction and Group, *F*(1,128) = 0.91 *p* = .343, η_p_^2^ = .01 or between Induction and Social desirability, *F*(1,128) = 0.00, *p* = .988, η_p_^2^ = .00. There was a between subjects effects of Social desirability on Joviality, *F*(1,128) = 17.68, *p* < .001, η_p_^2^ = .12, indicating that individuals with greater social desirability bias generally reported higher joviality. In sum, both positive MIPs successfully induced greater joviality, and although social desirability bias predicted higher joviality generally it did not interact with the positive MIPs.

#### Do the negative mood inductions increase sadness?

**Fig 2:**
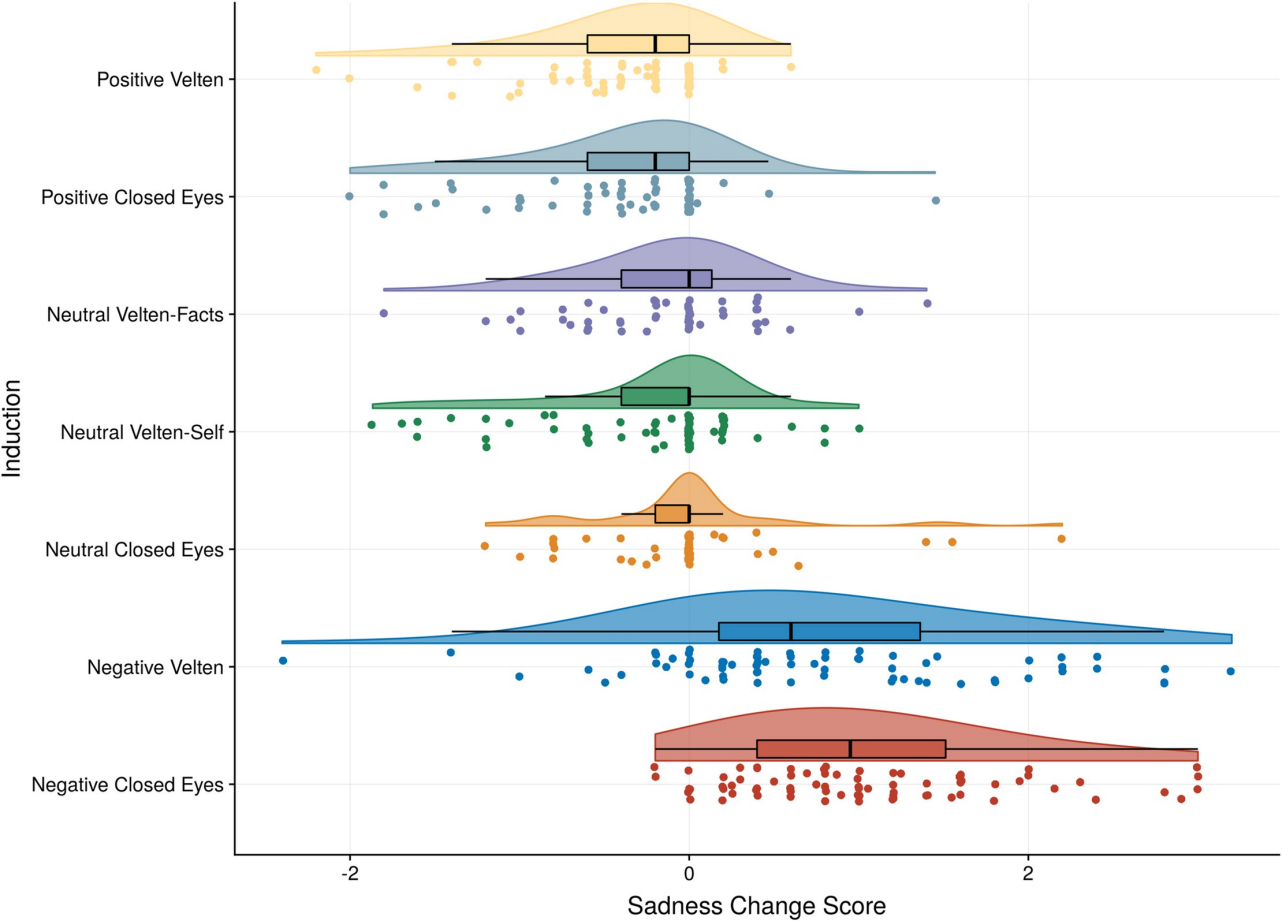
Illustration of sadness change scores across mood induction procedures. A raincloud plot showing the distributions of change scores in Sadness from pre to post-induction grouped by induction condition. The small dots below each raincloud show the observed scores of that condition (jittered). The lower and upper hinge on the boxplots represent the first and third quartile with the median in between, whereas the whisker lines extend to the most extreme values but no further than 1.5 × the interquartile range from the hinge. The raincloud shapes illustrate the distribution of observations based on Kernel density probability functions computed for each condition (scaled to have equal heights across conditions). To highlight “no change” from pre- to post induction, 0 is indicated with a vertical line.

The RM ANCOVA with the two negative Groups (Velten vs. closed eyes) as between-subjects factor, Induction (pre vs. post) as within-subjects factor, and Social desirability as a covariate indicated a main effect of the Induction, *F*(1,139) = 5.54, *p* = .020, η_p_^2^ = .04, as expected. The common language effect size indicates the likelihood that a person rates higher Sadness after the negative induction than prior to it is 83%, and the effect size was large (Hedges G_av_ = 0.98, 95% CI [0.78, 1.19]). The interaction between Induction and Group was not significant, *F*(1,139) = 3.89 *p* = .050, η_p_^2^ = .03. There was no significant interaction between Induction and Social desirability, *F*(1,139) = 3.20 *p* = .076, η_p_^2^ = .02, and no main effect of Social desirability on Sadness, *F*(1,139) = 1.20 *p* = .275, η_p_^2^ = .01. These conclusions did not change if we dropped any of the three potential outliers who had *reduced* sadness after the negative Velten induction (i.e., sadness change scores of -1 or lower; see Fig 2).

#### Do the neutral mood inductions change joviality or sadness?

Two RM ANCOVAs were performed with the three neutral Groups (closed eyes vs. Velten-Self vs. Velten-facts) as between-subjects factor, Induction (pre vs. post) as within-subjects factor, Social desirability as covariate, and Joviality and Sadness, respectively, as the DVs. Regarding Joviality, there was no significant main effect of Induction, *F*(1,168) = 0.09, *p* = .761, η_p_^2^ = .00, and no significant interaction between Induction and Group, *F*(2,168) = 0.47, *p* = .623, η_p_^2^ = .01, or Induction and Social desirability, *F*(1,168) = 3.18, *p* = .076, η_p_^2^ = .02. There was a main effect of Social desirability on Joviality, *F*(1,168) = 5.29, *p* = .023, η_p_^2^ = .03, indicating that individuals with greater bias reported greater joviality generally. It is noteworthy that when Social desirability is dropped from the model the main effect of Induction is significant, *F*(1,169) = 16.24, *p* < .001, η_p_^2^ = .09 (Hedges G_av_ = 0.27, 95% CI [0.13, 0.41]). That is, we could detect significant reductions in joviality after neutral inductions only when social desirability was unaccounted for.

With respect to Sadness, there was a significant main effect of Induction, *F*(1,168) = 5.27, *p* = .023, η_p_^2^ = .03, contrary to our expectations. The common language effect size indicated that the likelihood that a person report reduced sadness after the neutral induction than prior to it is 58%, and the effect size was very small (Hedges G_av_ = 0.15, 95% CI [0.05, 0.26]). There was no significant interaction between Induction and Group, *F*(2,168) = 1.94, *p* = .146, η_p_^2^ = .02, or between Social desirability and Induction, *F*(1,168) = 2.37, *p* = .126, η_p_^2^ = .01. To sum up, the neutral MIPs weakly reduced sadness.

#### Analyzing Velten and valence interaction effects

The last set of analyses tested interactions between Velten and valence. These analyses excluded the neutral Velten-facts group as there were no positive or negative facts groups of this kind. Thus, we performed a RM ANCOVA with Velten (Velten-self vs. Closed eyes) and Valence (Positive vs. Neutral vs. Negative) as the between-subjects factors, Induction (Pre vs. Post) as within-subjects factor, Social desirability as the covariate, and Joviality and Sadness as the DVs, respectively.

Regarding Joviality, there was no three-way interaction between Induction, Valence, and Velten, *F*(2,379) = 0.17, *p* = .841, η_p_^2^ = .00, and no two-way interaction between Induction and Velten, *F*(1, 379) = 0.99, *p* = .319, η_p_^2^ = .00. There was a large two-way interaction between Induction and Valence, *F*(2, 379) = 184.25, *p* < .001, η_p_^2^ = .49. We followed this effect up by analyzing difference scores in Joviality by valence condition. Joviality difference scores were greater in those completing the positive inductions (*M* = 0.62, *SD* = 0.54) than those in the neutral conditions (*M* = -0.25, *SD* = 0.75), with a large effect size, *t*(242) = 10.51, *p* < .001, Hedges *G*_*av*_ = 1.35, 95% CI [1.07, 1.63]. The common language effect size indicates an 83% chance that for a randomly selected pair of individuals the Joviality difference score of a person completing the positive induction will be higher than the score of a person completing the neutral condition. The Negative condition yielded negative difference scores in Joviality (*M* = -0.94, *SD* = 0.72), which was significantly more negative than the neutral condition, *t*(253) = 7.40, Hedges *G*_*av*_ = 0.93, 95% CI [0.67, 1.19], *p* < .001), with a 79% likelihood that a randomly selected person from the negative induction will have a larger drop in Joviality compared to a person from the neutral condition. In sum, compared to the neutral conditions, the positive and negative conditions induced large changes in joviality in the expected directions.

As for Sadness, there was no three-way interaction between Induction, Valence, and Velten, *F*(2,379) = 1.28, *p* = .279, η_p_^2^ = .01, but there was a two-way interaction between Induction and Velten, *F*(1, 379) = 5.74, *p* = .017, η_p_^2^ = .01. Follow up analyses indicated that there was a greater increase in Sadness in the Closed Eyes conditions (*M* = 0.27, *SD* = 0.94) than the Velten conditions (*M* = 0.05, *SD* = 0.91), with a 57% likelihood that for a randomly selected pair of individuals, the individual completing the Closed Eyes would have greater increase in Sadness than the one completing Velten, *t*(384) = 2.34, *p* < .020, Hedges *G*_*av*_ = 0.24, 95% CI [0.04, 0.44]. There was also a two-way interaction between Induction and Valence on Sadness, *F*(2, 379) = 121.84, *p* < .001, η_p_^2^ = .39. The negative condition increased Sadness (*M* = 0.90, *SD* = 0.93) more than the neutral condition (*M* = -0.13, *SD* = 0.60), *t*(253) = 10.18, *p* < .001, Hedges *G*_*av*_ = 1.28, 95% CI [1.01, 1.55], whereas the neutral had a smaller drop in Sadness than the positive (*M* = -0.40, *SD* = 0.57), *t*(242) = 3.63, *p* < .001, Hedges *G*_*av*_ = 0.47, 95% CI [0.21, 0.72]). There was also a two-way interaction between Social desirability and Induction on Sadness, *F*(1, 379) = 5.05, *p* = .025, η_p_^2^ = .01. Pearson correlations for pre and post, respectively indicated a weak, negative association between Social desirability and Sadness prior to induction, *r*(384) = -.14, *p* = .004, but no significant association after the induction, *r*(384) = -.08, *p* = .101. To conclude, sadness was generally higher in closed eyes conditions than Velten conditions, and social desirability was associated with sadness prior to induction, but these effects were very small. Large increases in Sadness were observed when comparing negative to neutral induction procedures, whereas positive inductions moderately reduced sadness compared to neutral inductions.

Finally, in an exploratory post-hoc analysis, we examined whether the device used when completing the study was related to the effectiveness of the induction methods, but we did not find evidence for any device effect. Specifically, we performed a RM ANCOVA with Device (Desktop vs. Laptop) and Valence (Positive vs. Neutral vs. Negative) as the between-subjects factors, Induction (Pre vs. Post) as within-subjects factor, Social desirability as the covariate, and Joviality and Sadness as the DVs, respectively. There was no significant main effect of Device nor interactions between Device and Valence or Induction on Joviality or Sadness, *p*s > .05. The effectiveness of the inductions did not significantly depend on whether participants used desktop or laptop device.

## General discussion

These studies update the Velten literature by adding neutral self-referential Velten statements and providing valence ratings of 80 individual statements, which can be used for quick, standardized mood inductions. As we ranked the valence ratings of each Velten statement, only four statements obtained incongruent ratings. Based on the results of Study 1, we developed sets of 15 statements per condition: positive, negative, neutral-self, and neutral-facts. Study 2 integrated these sets together with standard video and song clips to form combined online MIPs of 8 min that could induce relatively large effects in internet-based settings. Compared to neutral conditions, positive MIPs successfully induced large increases in joviality (and medium-sized decreases in Sadness). Likewise, compared to neutral conditions, the negative MIPs successfully induced large increases in Sadness (and large decreases in Joviality). These effect sizes compare favorably to those observed in a previous meta-analysis on internet-based MIPs (Ferrer et al., 2015), suggesting that these procedures can be useful for future online research on mood. There was no support for an advantage of Velten statements over closed eyes instructions when combined with music (and following a video clip), despite confirming the effectiveness of each Velten statement in Study 1 and selecting the most effective ones for Study 2.

The neutral MIPs weakly reduced Sadness and Joviality, but with Social desirability bias statistically controlled for, only the effect on sadness remained. Although we did not expect that these stimuli would change joviality and sadness, the reduced mood is arguably consistent with the notion of a neutral mood as a minimal affective state. Gasper defined neutral state in five ways: minimal affective, in-the-middle, deactivated, typical, or indifferent [41]. It is possible that our stimuli tap related but distinct neutral states. For instance, some of our Velten statements might induce a minimal affective state with low levels of both happiness and sadness (e.g., Some days are neither good nor bad), a state of indifference (e.g., Everyone seems to be going about their everyday routine just like me), in-the-middle state (e.g., If I think about it, things tend to even out for me), or typical state (e.g., Today is just an ordinary day). Future research could expand on these stimuli and explore whether these are distinct neutral states.

A few limitations of these studies need to be acknowledged. First, they relied on self-reported experiences. Although MIPs have shown effects on behavior, cognition, experience, judgment, and physiology [8], the effects observed in this study might partly be due to report biases. We tried to mitigate this risk by being transparent about our testing of different procedures and asking for honest self-reports. The fact that participants in internet-based research have no personal encounters with an experimenter might also reduce demand characteristics. Furthermore, the mood effects were large despite statistically controlling for individual differences in social desirability, which has been shown to be sensitive to demand characteristics manipulations [27]. Second, we measured mood immediately after the induction and can therefore not make any claims about the duration of the effects. It is possible that Velten and closed eyes inductions show divergent mood trajectories over time. Third, we constrained our affect measures to happiness and sadness. It is possible that the MIPs induce changes in other discrete emotions (e.g., surprise, confusion, serenity). Fourth, we estimated the effect size of combined MIPs as a step towards obtaining reliable, large mood effects online, but did not evaluate which component contributed the most or what duration is optimal. We assumed that combining video and music together with Velten or closed eyes instructions would be more effective than using them separately (e.g., cartoon videos might ease the viewer into a targeted mood, making them more susceptible to the suggestive Velten or closed eyes instructions to get deeper into the targeted mood—similar to how becoming absorbed in appealing stimuli might increase the effectiveness of hypnotic suggestions [42]). However, we cannot evaluate which components were effective, whether there were additive or interactive effects, or whether shorter MIPs would yield similar effect sizes. Addressing those questions was beyond the scope of this paper, but it would be informative to develop MIPs that are more time effective and involve fewer possibly confounding factors. A possible strength with combined MIPs is that limitations due to specific components of the inductions can be mitigated by the other components and that they might therefore be effective on a broader population.

To sum up, we developed 8 min induction procedures that can be used in online research to induce large changes in happiness and sadness compared to a neutral induction. Although there were weak-to-medium associations between mood and social desirability bias, the latter could not account for the large effects of the positive and negative induction procedures.

## Supporting information

S1 File S1_Pre-registration. Study 2 pre-registration.
(PDF)

S2 File S2_Dataset_Study_1. SPSS dataset study 1.
(SAV)

S3 File S3_Dataset_Study_2. SPSS dataset study 2.
(SAV)
