# Supplementary material for: Online validation of combined mood induction procedures
Source: PLoS One. 2019 Jun 4;14(6):e0217848. doi: 10.1371/journal.pone.0217848 (PMC6548374; doi:10.1371/journal.pone.0217848)
Supplement: S1 File — Study 2 pre-registration. (PDF) [file pone.0217848.s001.pdf]

ASPREDICTED

Testing Combined Mood Induction Procedures (Velten/film/music/closed-eyes) (#12790)

Author(s)
David Marcusson-Clavertz (Lund University) - david.marcusson-clavertz@psy.lu.se
Oscar Kjell (Lund University) - oscar.kjell@psy.lu.se
Stefan Persson (Lund University) - stefan.persson@psy.lu.se
Etzel Cardeña (Lund University) - etzel.cardena@psy.lu.se

Created: 07/20/2018 03:43 AM (PT)
Public: 05/16/2019 04:16 PM (PT)

1) Have any data been collected for this study already?
No, no data have been collected for this study yet.

2) What's the main question being asked or hypothesis being tested in this study?
1) Do combined, online mood induction procedures (MIPs; film clips followed by songs with Velten or Closed eyes) induce the intended moods?
a) Do positive MIPs (with Velten-Self or Closed eyes) increase happiness? We expect they do.
b) Do negative MIPs (with Velten-Self or Closed eyes) increase sadness? We expect they do.
c) Do neutral MIPs (with Velten-Facts or Self or Closed eyes) change happiness or sadness? We do not expect they do.

2 Do Velten and Closed Eyes conditions differ in their efficacy (when combined with film clips and music)? This is exploratory, we do not have any expectation.
a) Do positive MIPs with Velten and Closed eyes differ in changing happiness?
b) Do negative MIPs with Velten and Closed eyes differ in changing sadness?
c) Do neutral MIPs with Velten-Self, Velten-Facts, and Closed eyes differ in changing happiness and sadness?

3) Describe the key dependent variable(s) specifying how they will be measured.
Happiness = Joviality scale of the PANAS-X (8 items). Sadness = Sadness scale of the PANAS-X (5 items).

4) How many and which conditions will participants be assigned to?
Repeated measure (2 levels): pre and post MIP. Between-subjects Groups (7 groups):
2 Positive groups with Velten-Self or Closed-Eyes
2 Negative groups with Velten-Self or Closed-Eyes
3 Neutral groups with Velten-Facts, Velten-Self, or Closed-Eyes

5) Specify exactly which analyses you will conduct to examine the main question/hypothesis.
We will include the Social Desirability Scale (16 items) as a covariate in all analyses.
1a) and 2a) (Positive MIPs). An Repeated Measures (RM) ANOVA with Induction (pre vs. post) as within-factor and group (Positive Velten vs. Closed-eyes) as between-factor. 1a is examined with the main effect of the induction. 2a is examined with the interaction induction*group. The dependent variable (DV) is happiness.

1b) and 2b) (Negative MIPs). An RM ANOVA with Induction (pre vs. post) as within-factor and group (Negative Velten vs. Closed-eyes) as between-factor. 1b is examined with the main effect of the induction. 2b is examined with the interaction induction*group. The DV is sadness.

1c) and 2c) (Neutral MIPs). An RM ANOVA with Induction (pre vs. post) as within-factor and group (Neutral Velten-Facts vs. Velten-Self vs. Closed-eyes) as between-factor. 1c is examined with the main effect of the induction. 2c is examined with the interaction induction*group. The RM ANOVAs will have happiness and sadness as the DVs.

6) Describe exactly how outliers will be defined and handled, and your precise rule(s) for excluding observations.
We will first exclude participants that answer any of the 3 control items incorrectly.
We will then exclude multivariate outliers using a Mahalanobis distance cutoff of p less than .001.

7) How many observations will be collected or what will determine sample size? No need to justify decision, but be precise about exactly how the number will be determined.
We will collect data from 500 individuals.

8) Anything else you would like to pre-register? (e.g., secondary analyses, variables collected for exploratory purposes, unusual analyses planned?)
We will perform secondary analysis with 2 (pre vs. post) *2 (velten vs. closed eyes) *3 (positive vs. neutral vs. negative) RM ANOVAs to compare valence conditions across DVs, excluding the Neutral-Facts. For an unrelated project we will administer the Spontaneous and Deliberate Mind Wandering scales to explore if MIPs influence mind wandering report.
